# Effects of prefrontal theta burst stimulation on neuronal activity and subsequent eating behavior: an interleaved rTMS and fNIRS study

**DOI:** 10.1093/scan/nsab023

**Published:** 2021-02-22

**Authors:** Idris Fatakdawala, Hasan Ayaz, Adrian B Safati, Mohammad N Sakib, Peter A Hall

**Affiliations:** School of Public Health and Health Systems, University of Waterloo, Waterloo, CA, ON, Canada; School of Biomedical Engineering, Science and Health, Drexel University, Philadelphia, PA, USA; Department of Psychology, College of Arts and Sciences, Drexel University, Philadelphia, PA, USA; Drexel Solutions Institute, Drexel University, Philadelphia, PA, USA; Department of Family and Community Health, University of Pennsylvania, Philadelphia, PA, USA; Center for Injury Research and Prevention, Children’s Hospital of Philadelphia, Philadelphia, PA, USA; School of Public Health and Health Systems, University of Waterloo, Waterloo, CA, ON, Canada; School of Public Health and Health Systems, University of Waterloo, Waterloo, CA, ON, Canada; School of Public Health and Health Systems, University of Waterloo, Waterloo, CA, ON, Canada; Centre for Bioengineering and Biotechnology, University of Waterloo, Waterloo, Canada; Department of Psychology, University of Waterloo, Waterloo, ON, Canada

**Keywords:** eating, rTMS, fNIRS, dmPFC, dlPFC, cortex

## Abstract

The dorsolateral prefrontal cortex (dlPFC) and dorsomedial prefrontal cortex (dmPFC) are both important nodes for self-control and decision-making but through separable processes (cognitive control *vs* evaluative processing). This study aimed to examine the effects of excitatory brain stimulation [intermittent theta burst stimulation (iTBS)] targeting the dlPFC and dmPFC on eating behavior. iTBS was hypothesized to decrease consumption of appetitive snack foods, via enhanced interference control for dlPFC stimulation and reduced delay discounting (DD) for dmPFC stimulation. Using a single-blinded, between-subjects design, participants (*N *= 43) were randomly assigned to one of three conditions: (i) iTBS targeting the left dlPFC, (ii) iTBS targeting bilateral dmPFC or (iii) sham. Participants then completed two cognitive tasks (DD and Flanker), followed by a bogus taste test. Functional near-infrared spectroscopy imaging revealed that increases in the medial prefrontal cortex activity were evident in the dmPFC stimulation group during the DD task; likewise, a neural efficiency effect was observed in the dlPFC stimulation group during the Flanker. Gender significantly moderated during the taste test, with females in the dmPFC showing paradoxical increases in food consumption compared to sham. Findings suggest that amplification of evaluative processing may facilitate eating indulgence when preponderant social cues are permissive and food is appetitive.

## Introduction

The prefrontal cortex (PFC) contains several important nodes of the executive control network, supporting a variety of functions including inhibitory control and evaluative processing (MacPherson *et al.*, 2002; Siddiqui *et al.*, 2008). Dietary self-control is partially dependent on the lateral PFC, as it is thought to facilitate suspension of prepotent approach behaviors to high-caloric foods in the interests of restraint goals (Hall, 2016; Lowe *et al.*, 2019). For this reason, it has been hypothesized that the extent to which indulgent eating occurs in permissive contexts may be partially dependent on the integrity of the lateral PFC as well as its functional connectivity with other brain regions involved in value and salience processing (Hall, 2016; Donofry *et al.*, 2019; Lowe *et al.*, 2019). There is an accumulating body of literature linking attenuated dorsolateral prefrontal cortex (dlPFC) function with indulgent eating (Brooks *et al.*, 2013; Han *et al.*, 2018; Schmidt *et al.*, 2018; F. Chen *et al*., 2018; Donofry *et al.*, 2019). Likewise, some evidence indicates that indulgent food consumption is linked with attenuated function of these same cortical nodes (Kalon *et al.*, 2016; R. Chen *et al*., 2018), supporting the possibility of a reciprocal relationship between PFC function and obesity (Lowe *et al.*, 2019). A meta-analysis of experimental studies examining the effects of non-invasive brain stimulation methods revealed that excitatory stimulation of the dlPFC decreases, and suppressive stimulation increases, indulgent food consumption (Hall *et al.*, 2017; Lowe *et al.*, 2017).

In the clinical trial literature, multi-session repetitive transcranial stimulation (rTMS) intervention studies have shown that excitatory stimulation of the left dlPFC reduces cravings for appetitive foods among those with eating disorders (Uher *et al.*, 2005; Van Den Eynde *et al.*, 2010). Likewise, multi-session excitatory rTMS targeting the left dlPFC was found to be effective in enhancing weight loss and decreasing food intake in obese patients in at least one recent study (Kim *et al.*, 2019). On the other hand, in the experimental literature, continuous theta burst stimulation (cTBS; a suppressive variant of rTMS) targeting the left dlPFC results in amplified cravings and consumption of similar foods when applied in single-session format (Lowe *et al.*, 2014, 2018b). Meta-analyses examining the effects of non-invasive brain stimulation techniques reveal medium effect sizes on food cravings (Jansen *et al.*, 2013; Lowe *et al.*, 2017) and consumption outcomes (Hall *et al.*, 2017; Song *et al.*, 2019), both in favor of active over sham stimulation. Such findings are similar to those observed for neuromodulation studies involving other types of cravings (Jansen *et al.*, 2013).

Given that cTBS targeting the dlPFC reliably alters inhibitory task performance in theorized directions (Lowe *et al.*, 2018a), it is reasonable to posit that excitatory stimulation effects on eating might be mediated through improved performance on cognitive tasks that load heavily on inhibitory functions. However, the specific PFC subregion targeted is an important consideration. Like inhibitory control, evaluative processing and self-relevance processing—supported by the medial subregions (mPFC)—are also implicated in both indulgent eating (Hare *et al.*, 2011; Rangel, 2013; Dietrich *et al.*, 2016; Berkman *et al.*, 2017; R. Chen *et al*., 2018; Hall, 2019) and self-regulatory processes in relation to tempting stimuli (Cho *et al.*, 2015; Hall, 2019). Indeed, functional connectivity between the mPFC and other brain regions is altered following gastric bypass surgery, one of the more successful approaches to radical weight reduction (Li *et al.*, 2018).

For these reasons, stimulation of the mPFC might also impact indulgent eating by altering evaluative processing of food-related stimuli or affecting the link between value processing and eating behavior regulation. The directionality of anticipated effects on eating indulgence is clear for the lateral PFC, given its role in inhibitory control; however, there is less certainty about the mPFC, given that it is implicated in value computation and mind wandering, both of which could sway behavior both toward or away from eating indulgence, depending on the preponderance of active value signals or sensory stimuli (i.e. in favor of indulgent consumption or away from it) in the eating environment. In either case, the mPFC, broadly speaking, appears to be implicated in value signal processing in a manner that is broadly relevant for self-regulation of eating behavior (Hare *et al.*, 2011; Rangel, 2013; Berkman *et al.*, 2017).

In this context, the purpose of the current study was to examine the role of two PFC subregions—the dlPFC and dorsomedial prefrontal cortex (dmPFC)—in eating behavior, as mediated through either inhibitory or evaluative processing. Functional near-infrared spectroscopy (fNIRS; Ferrari and Quaresima, 2012; Curtin and Ayaz, 2018; Pinti *et al.*, 2020) was used to examine task-related neural activity in both PFC subregions following rTMS (Curtin *et al.*, 2019b), and eating indulgence was assessed using a bogus taste test paradigm involving calorie-dense snack foods (Robinson *et al.*, 2017). Given research precedent, we anticipated that excitatory stimulation targeting the lateral PFC would enhance neural activity—as indexed by regional oxygenated hemoglobin concentrations, assessed using fNIRS—during Flanker performance (i.e. a sign of adaptive network engagement). Conceptually, performance on interference paradigms such as the Flanker is hypothesized to rely on the behavioral inhibition facet of executive function quite centrally, thereby implicating the dlPFC. Likewise, in terms of research precedent reflected by meta-analytic reviews, it was expected that neuromodulation targeting the dlPFC should affect inhibitory task performance (Lowe *et al.*, 2018a), as well as indulgent eating behavior itself (Hall *et al.*, 2017).

Hypotheses regarding dmPFC stimulation were more tentative, given the less certain effect of excitatory TBS protocols on neural activity in this PFC subregion and the potentially agnostic role of evaluative processing in self-regulation of indulgent eating. In terms of cognitive task paradigms involving self-relevant evaluative processing (i.e. considering multiple contingencies that might affect the self, and weighing each on a temporal plane), delay discounting (DD) tasks are prominent and often implicated in evaluative aspects of eating behavior (Epstein *et al.*, 2010; Koffarnus and Bickel, 2014). Prior studies have shown that DD is intimately connected with mPFC function (Cho *et al.*, 2015; Owens *et al.*, 2017, 2019) and so this task was chosen to elicit task-related activity involving evaluative processing.

Finally, given that gender differences exist in food cravings (Hallam *et al.*, 2016), along with possible differences in brain stimulation effects between younger and older adults (Oberman and Pascual-Leone, 2013; Gutchess, 2014), we also examined for participant gender and age group as moderators for our experimental effects.

## Methods

### Participants

A sample of 43 participants was recruited for the study. To assess the differences in the effects of intermittent theta-burst stimulation (iTBS) by age, the recruitment was stratified as follows: 22 younger adults and 21 middle-to-older aged adults (‘older adults’). Younger adults were between 18 and 30 years of age and were all recruited from advertisements placed around campus. The older adults were between 40 and 75 years of age and were recruited from campus, an aging participant pool as well as from local community centers. All eligible participants were right-handed, neurologically healthy and naïve to TMS; prior to participation individuals were screened for any physical and neurological conditions that would contraindicate rTMS, using a standard screening form (Rossi *et al.*, 2009). Participants with food intolerances or health conditions (e.g. diabetes) that would preclude normative participation in the taste test were also excluded. Following explanation of risks and benefits of participation, electronic informed consent was obtained from all participants prior to the start of the study. In exchange for their participation, each participant received a $25 gift card as reimbursement for their time. The study was reviewed by and received ethics clearance from the University of Waterloo research ethics committee.

### Procedure

 The study employed a single-blinded between-subject design in which participants were randomly assigned to one of the three conditions: iTBS targeting the dlPFC, iTBS targeting the dmPFC and sham iTBS targeting the vertex. Each study session was conducted at 10:00 am–11:30 am (morning session) or 1:30 pm–3:00 pm (afternoon session) from Monday to Friday. Participants were asked to refrain from eating or consuming any caffeinated beverages 3 h prior to the study; adherence to these requirements was checked with the completion of the consent and screening forms. All computer tasks were presented using the Inquisit (Millisecond Software) on a 27-inch monitor. Prior to commencing the computer tasks, participants were asked to follow the instructions that were presented on the monitor and respond as quickly and accurately as possible while completing each cognitive task. The ambient lighting and temperature conditions were stable across all participants.

 Following consent procedures, resting motor threshold (RMT) was determined followed by active or sham stimulation, as randomly assigned. Ten minutes post-stimulation, participants were asked to complete the two computerized cognitive tasks in the following order: three blocks of the DD task, followed by four blocks of the flanker inhibition task. Between each block, there was a 15 s rest period. While performing these tasks, changes in blood oxygenation levels were measured using the fNIRS protocol (see below). Following the completion of the cognitive tasks—∼30 min post-stimulation (when iTBS excitatory effects would be maximal)—participants were given the opportunity to sample five different calorie-dense snack foods under the guise of examining the relationship between brain function and taste perception. Change in the weight of the food from pre-to-post tasting was measured surreptitiously to quantify the amount of food that was consumed.

### Brain stimulation protocol

 iTBS targeting the left dlPFC was administered using a 75 mm diameter figure-8 coil (Magventure MCF-B65), while the iTBS stimulation for the bilateral dmPFC was administered using a 97 mm figure-8 coil (Magventure MCF-B70), both which were connected to a Magventure Mag Pro x100 biphasic stimulation unit. Sham stimulation was delivered with a placebo version of the MCF-B65 (i.e. Magventure MCF-P-B65), targeting the vertex. The iTBS stimulation intensity to the left dlPFC was individually calibrated based on each participant’s RMT, as per standard practice. The RMT was defined as the lowest stimulation intensity required to induce a motor evoked potential in the right abductor pollicus brevis muscle (the right thumb muscle) of >50 µV peak-to-peak amplitude, respectively, in 5/10 consecutive trials of stimulating the motor cortex. In order to guide coil placement for stimulating both the motor cortex (for determining the RMT) and the cortical target regions of interest, an EEG cap with electrodes arranged in the international 10–20 system fitted according to standard anatomical landmarks. The determination of the RMT was made using the C3 position to approximate the motor cortex; the iTBS stimulation site for the left dlPFC was approximated by the F3 electrode position. Finally, the iTBS stimulation site for the bilateral dmPFC was approximated as 2/3 of the distance from the nasion to the vertex, as per prior research precedent (Berkers *et al.*, 2017).

 Following RMT determination, the intensity for left dlPFC stimulation was set at 80% of the RMT. Stimulation consisted of triplet stimuli applied in the theta burst pattern (three 50 Hz pulses repeated at a frequency of 5 Hz); TBS was applied for 2 s for every 10 s period (i.e. 2 s of TBS followed by 8 s of rest), for a duration of 190 s, totaling 600 stimuli (Huang *et al.*, 2005). Participants assigned to the sham iTBS condition received a procedure identical to that described above using the placebo version of the same coil (MCP-P-B65) targeting the vertex (Cz position) instead. The MCF-P-B65 contains a coating that blocks 80% of the stimulation intensity delivered by the coil, which is otherwise visually and mechanically identical to the MCF-B65. In the dmPFC stimulation condition, stimulation intensity was set at a fixed intensity of 30% of the maximal stimulator output in keeping with prior research using thisstimulation site (Berkers *et al.*, 2017).

### fNIRS **protocol**

fNIRS is a non-invasive, optical neuroimaging technique that uses near-infrared (NIR) light sources and detectors to quantify changes in blood oxygenation levels within cortical brain tissues following neuronal activation (Ferrari and Quaresima, 2012; Curtin and Ayaz, 2018; Pinti *et al.*, 2020). In order to measure cerebral activation, fNIRS relies on a hemodynamic response, which produces a relative increase in oxygenated hemoglobin (HbO) and decrease deoxygenated hemoglobin (HbR) during neuronal activity (Ayaz *et al.*, 2019; Pinti *et al.*, 2020). Because HbO and HbR absorb NIR light at different wavelengths, fNIRS is able to take advantage of chromophoric features of hemoglobin to detect changes in brain activation.

For this study, fNIR Devices 203C imaging system was used to quantify regional oxygen saturation in the PFC. The equipment montage consisted of a headband with a sensor pad, which was embedded with 4 LED light sources and 10 light detectors, joined to create 16 channels (plus two short channels). Two wavelengths of light, at 730 nm (for HbR) and 850 nm (for HbO), were measured using the COBI Studio software (Ayaz *et al.*, 2011). Signal processing procedures were conducted using *fnirSoft* Professional (Version 4.11) (Ayaz *et al.*, 2012). For each participant, raw fNIRS light intensity data were low-pass filtered with a finite impulse response, linear phase filter with order 20 and cut-off frequency of 0.1 Hz to attenuate the high-frequency noise, respiration and cardiac cycle effects (Ayaz *et al.*, 2010). Each participant’s data were checked for any potential saturation (when light intensity at the detector was higher than the analog-to-digital converter limit) and motion artifact contamination by means of a coefficient of variation-based assessment (Ayaz *et al.*, 2010). fNIRS data for each task block were extracted using beginning and end timings based on the experiment, and hemodynamic changes for each of 16 channels during each trial block were calculated separately using the Modified Beer–Lambert Law with respect to the local baseline at the beginning of the respective block. The hemodynamic response at each channel was then averaged across time for each trial block to provide mean hemodynamic response at each channel for each block to be used in statistical analysis.

### Cognitive tasks

#### DD task.

Participants were asked to complete a variant of the DD paradigm described by Koffarnus and Bickel (2014). The task is an adjusting DD task, which uses the concept of ED50 (Effective Delay 50%), to determine a delay that is effective in discounting the value of the delayed reinforcer by 50% (Koffarnus and Bickel, 2014). Participants were presented with three blocks, with the option of choosing between two options: a fixed larger commodity for which the delay was adjusted from trial-to-trial verses a fixed smaller commodity that was immediately available. The magnitude of the immediately available option for each block was set at half of the delayed option (Block 1: $5 *vs* $10, Block 2: $500 *vs* $1000, Block 3: $500 000 *vs* $1 000 000). Each block consisted of five trials. The first-choice trial for each block was always set with the larger commodity delayed at 3 weeks. For subsequent trials, the delay for the larger option was adjusted depending on the participant’s previous choice; delay was adjusted up if the delayed choice was chosen or down if the immediate choice was chosen on the previous trial. The degree of discounting (*k* value) was determined at the end of last trial from the respective ED50 value (the delay at which the preference switches from the larger delayed option to the immediately available option, that is half in magnitude) obtained from Koffarnus and Bickel (2014). The *k* value was then computed using the following modified hyperbolic equation (see Yoon and Higgins, 2008; Koffarnus and Bickel, 2014):

$$\begin{equation*}{\mathbf{\it{ED}50}}\,\,{\mathbf{=}}\,\,{\mathbf{1/\it{k}}}\end{equation*}$$

Conveniently, this suggests that the ED50 value is simply the inverse of the rate of discounting (1/*k*). A smaller *k* value is taken to indicate the relative absence of discounting, thereby implying a preference for delayed rewards. A higher *k* value is indicative of a strong discounting rate, thereby implying a preference for immediate rewards.

#### Flanker task.

Participants were asked to complete a modified version of the Eriksen Flanker task, which was used to measure behavioral inhibition. In this task, participants were presented with a stimulus consisting of a set of seven letters and were asked to make directional responses to the letter in the center (the target stimuli) arranged among an array of flanking letters (non-target stimuli), by pressing the corresponding keyboard key that is assigned to target stimuli. The target letters ‘H’ and ‘K’ were assigned to either the ‘A’ or ‘D’ keyboard key, while the target letters ‘S’ and ‘C’ were assigned to the alternative key. Participants were presented with two conditions: 1) congruent noise condition, in which the target letter was flanked by the letter corresponding to the same keyboard key response (i.e. HHHKHHH or CCCSCCC), and 2) incongruent noise condition, in which the target letter was flanked by the letters assigned to the other keyboard key response (i.e. CCCHCCC or HHHSHHH). Participants were required to make their response to the target letter as quickly and accurately as possible. The task began with a practice block, (60 trials of incongruent + congruent), followed by four blocks (two blocks of each condition), completed in the following order: 50 trials of the congruent task, 75 trials of the incongruent task, 50 trials of the congruent task and 50 trials of the incongruent task. A coding error led to a larger number of trials in the first incongruent block than others. However, all subsequent analysis was unaffected by this error. Flanker interference score was calculated by taking the difference of the average latency of the correct trials in the congruent noise condition from the average latency of correct trials in the incongruent noise condition. A higher score, therefore, reflected a worse performance on the task.

#### Taste test and food ratings.

Bogus taste test paradigms are commonly used in the eating literature as a reliable and valid metric of consumption; quantity consumed is positively associated with food palatability, level of hunger (Robinson *et al.*, 2017) and responsive to acute manipulations of executive function using TMS targeting the left dlPFC (Lowe *et al.*, 2018b). In the current version of the paradigm, participants were presented an array of five calorie-dense snack foods (three types of Pringles potato chips and two types of Belgian chocolate balls; milk chocolate and salted chocolate). Participants were given 15 min for the task, during which they were asked to complete a 7-item self-reported taste ratings questionnaire for each food item presented. The first reporting item had participants select from a list of 25 descriptive terms on the texture of the food that they had sampled. The remaining six items had each participant indicate their sensory experience (appealing, salty, sweet, greasy and healthy) and overall rating of the food on a scale of 1 to 10; (response scale: 1 = ‘Not at all [appealing, salty, etc.]’; 5 = ‘Moderately [appealing, salty, etc.]’; 10 = ‘Very [appealing, salty, etc.]*’* Prior to the task, participants given a verbal cue ‘eat as much as you would like while making your taste ratings’, under the guise that the main purpose of the task was to explore TMS effects on sensory processing. The experimental foods were surreptitiously weighted before and after the taste test, where the difference (amount of food consumed) was recorded (in grams) as the primary metric of food consumption. A total score and separate food type (i.e. sweet *vs* salty) scores were calculated.

### Statistical analyses

Descriptive statistics were computed to examine the distribution of each of the continuous outcome variables of interest: (i) food consumption, (ii) Flanker interference scores and (iii) averaged DD *k* values by treatment condition. Boxplots were generated to visually examine the characteristics of each variable distribution and identify any outliers. All the outcome variables in the dataset were subject to winsorization to limit the effects of extreme outliers. Four outliers were identified for the food consumption variable: two below the 5th percentile value and two above the 95th percentile value, which were than replaced with an assigned percentile value. In addition, granular analyses were conducted to compare differences in type of food consumed (salty *vs* sweet). One participant’s Flanker score was dropped because of accuracy scores below chance level. Four additional scores were replaced with winsorized values. The DD variable (*k*) was averaged across the three trials, to obtain an average discounting rate for each participant. Because the variable displayed significant skewness, *k* scores were then subject to a Log10 transformation in order to improve the distributional properties. Four values were replaced with their assigned winsorized percentile values. Next, univariate general linear models were employed to examine the effects of the stimulation condition (active dlPFC stimulation and active mPFC stimulation and sham stimulation) on the candidate outcome variables (i.e. food consumption, Flanker performance and DD). Age category and gender were examined as a moderator of treatment effects. Planned comparisons were conducted using independent *t*-tests.

The fNIRS data from the cognitive tasks were analyzed in the following manner: **Flanker Task**: Following the calculation of the mean hemodynamic response for each trial block at each channel, individual hemodynamic responses for the congruent and incongruent blocks were combined in order to obtain a mean hemodynamic response for each condition, at each channel. Next, the Flanker oxy-hemoglobin (OxyHb) contrast effect (mean hemodynamic response for the incongruent condition—mean hemodynamic response for congruent condition; see Supplementary material) was calculated at each channel and was subjected to a one-way Analysis of Variance (ANOVA), to test for any hypothesized group differences (active iTBS and sham iTBS) on the contrast. **DDTask**: Following the calculation of the mean OxyHb response for each trial block, the three-DD mean OxyHb responses were combined together, to obtain a mean hemodynamic response for the task, at each channel (see Supplementary material). The response at each channel was subject to a one-way ANOVA, to test for any hypothesized group differences (active iTBS and sham iTBS) on the DD task.

Differences between stimulation conditions across channels are illustrated using a heat map and a 3D anatomical overlay from corresponding *F*-values and *P-*values for each channel. Each channel was subject to winsorization prior to running the GLM’s.

## Results

The three stimulation groups did not differ with respect to age (*F*(2,40) = 0.043, *P* = 0.953), gender (*X^2^= *0.171, *P *= 0.918), body mass index (BMI) (*F*(2,40) = 1.601, *P* = 0.214) or time since last meal *(F*(2,40) = 1.724, *P* = 0.191; Table 1). No significant differences in RMT were found between the two calibrated stimulation conditions (dlPFC and sham; (*t*(1,28) = 0.323, *P* = 0.749)). Likewise, taste ratings did not differ among the three groups with respect to overall appeal (*F*(2,40) = 0.671, *P* = 0.517), saltiness (*F*(2,40) = 0.159, *P* = 0.854), sweetness (*F*(2,40) = 0.651, *P* = 0.546), greasiness (*F*(2,40) = 1.811, *P* = 0.177), healthiness (*F*(2,40) = 0.460, *P* = 0.634) or globally palatability (*F*(2,40) = 0.566, *P* = 0.572); this confirms that stimulation did not influence sensory processing of the foods presented in the taste test.

**Table 1.** Mean (SD) for demographic variables by treatment condition

	*dlPFC condition*	*mPFC condition*	*sham condition*	*Overall*
	*(n = 16)*	*(n = 13)*	*(n = 14)*	*(n = 43)*
*Age (years)*	44.87 (25.69)	42.38 (24.26)	44.86 (26.43)	44.12 (24.93)
*Gender*	11 Female	8 Female	9 Female	28 Female
	5 Male	5 Male	5 Male	15 Male
*Age category*	8 young adults	7 young adults	7 young adults	22 young adults
	8 older adults	6 older adults	7 older adults	21 older adults
*BMI*	26.08 (4.27)	25.82 (3.53)	23.89 (2.63)	25.29 (3.63)
*Last meal (hours)*	8.55 (4.65)	7.50 (4.52)	5.71 (3.20)	7.31 (4.26)
*iTBS intensity (% of max output)*	46.87 (5.78)	30.00*	46.14 (6.64)	41.53 (9.21)

* *Fixed* *intensity.*

### Neural response to iTBS

#### Flanker OxyHb contrast.

**Fig. 1.**
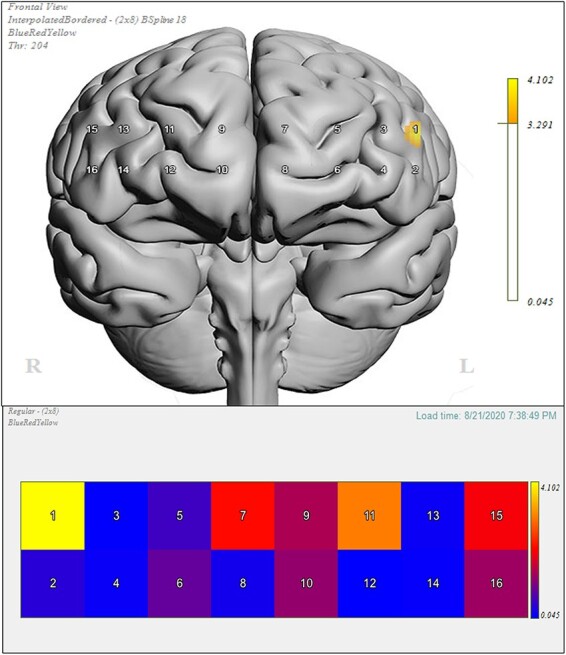
Left: heat map of channels 1–16 illustrating OxyHb concentration change from baseline between active iTBS (targeting the left dlPFC) and sham iTBS (targeting the vertex) on the Flanker contrast effect (incongruent − congruent). Color coding was represented by the strength of *F* and *P* values; warmer colors represent stronger differences on the contrast between active and sham; darker shades of blue represent weak to no differences. Right: 3-D anatomical overlay of the significant differences between active and sham. This figure was generated using fnirSoft (Version 4.11).

Channel-by-channel group differences between active and sham stimulation on the Flanker OxyHb contrast effect (mean incongruent and mean congruent) are displayed using a heat map (Figure 1, bottom), using corresponding *F* and *P* statistic values. The differences between the stimulation groups on the contrast are also overlaid upon a 3D greyscale anatomical brain (Figure 1, top).

**Fig. 2.**
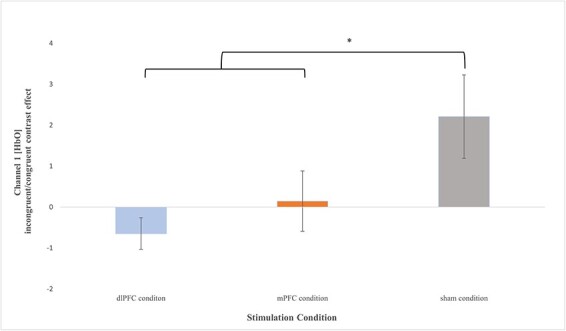
Means (±SE) for channel 1 Flanker OxyHb contrast effect for each stimulation condition; (A) dlPFC condition (*M* = −0.646, *SE* = 0.386), (B) mPFC condition (*M* = 0.146, *SE* = 0.735) and (C) sham condition (*M* = 2.21, *SE* = 1.02). *: *P* < 0.05.

 A significant Flanker OxyHb contrast effect was observed for channel 1 (*F*(2,29) = 4.102, *P* = 0.027, ${\eta _p}$^2^ = 0.221) when comparing the active iTBS conditions to sham. Specifically, those in active iTBS conditions had significantly lower OxyHb response (dlPFC = *M *= −0.646, *SE* =* *0.386, mPFC condition = *M* =* *0.146, *SE* = 0.735) on the contrast than those in the sham condition (*M* =* *2.21, *SE* = 1.02) during the Flanker task. Variable means for all stimulation conditions are depicted in Figure 2 below.

Planned comparisons for channel 1 indicated that compared to the sham condition, those in the dlPFC stimulation condition had a significantly lower OxyHb response (*t* (1,21) = −2.706, *P* = 0.013, 95% CI [−5.06, −0.662], *g* = −1.088). On the other hand, compared to the sham condition, those in mPFC stimulation condition had no significant differences in OxyHb response in channel 1 (*t* (1,18) = −1.576, *P* = 0.133, 95% CI [−4.83, 0.693], *g* = −0.677). Likewise, no significant differences on the OxyHb were observed between the dlPFC and mPFC conditions for channel 1 (*t* (1,19) = −1.023, *P* = 0.319, 95% CI [−2.41, 0.828], *g* = −0.433).

#### Moderation analyses.

Two-way ANOVAs (stimulation × age category and stimulation × gender) were generated for channel 1 in order to determine if the OxyHb contrast effect was further moderated by age or gender.

With respect to OxyHb concentrations for the Flanker contrast effect, the two-way (stimulation × age category) ANOVA revealed a significant main effect of stimulation (*F*(2,26) = 3.699, *P* = 0.039, ${\eta _p}$^2^ = 0.229), but no significant main effect of age category (*F*(1,26) = 3.052, *P* = 0.092, ${\eta _p}$^2^ = 0.105). The interaction between stimulation condition and age category was not significant (*F*(2,26) = 0.749, *P* = 0.483, ${\eta _p}$^2^ = 0.054).

**Fig. 3.**
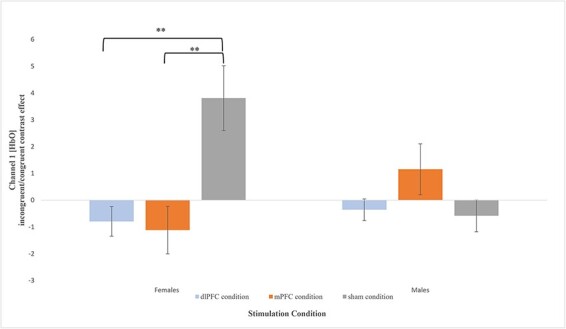
Means (±*SE*) for channel 1 Flanker OxyHb contrast effect by gender for each treatment condition; (i) Females: (A) dlPFC condition (*M* = −0.791, *SE* = 0.553), (B) mPFC condition (*M* = −1.114, *SE* = 0.886) and (C) sham condition (*M* = 3.814, *SE* = 1.212); (ii) Males: (A) dlPFC condition (*M* = −0.356, *SE* = 0.407), (B) mPFC condition (*M* = −1.154, *SE* = 0.950) and (C) sham condition (*M* = −0.582, *SE* = 0.593). **: *P *< 0.01.

With respect to OxyHb concentrations for the Flanker contrast effect, the two-way (stimulation × gender) ANOVA revealed a marginal main effect of stimulation (*F*(2,26) = 3.047, *P* = 0.065, ${\eta _p}$^2^ = 0.190) but no significant main effect of gender (*F*(1,26) = 0.544, *P* = 0.467, ${\eta _p}$^2^ = 0.021). The interaction between stimulation condition and gender was significant (*F*(2,26) = 6.674, *P* = 0.005, ${\eta _p}$^2^ = 0.339); the pattern of means suggests differences in the effects of the stimulation on the OxyHb contrast between males and females for channel 1. Variable means for all stimulation conditions by gender for channel 1 are presented in Figure 3 below.

Planned comparisons indicated that compared to the sham condition (*M* *= *3.814*, SE* = 1.212), females in the dlPFC stimulation condition (*M* = −0.791, *SE* = 0.553) had a significantly lower OxyHb contrast effect (*t* (1,13) = −3.612, *P* = 0.003, 95% CI [−7.39, −1.85], *g* = −1.759).

On the other hand, compared to the sham condition (*M* = −0.582, *SE* = 0.593), males in the dlPFC stimulation condition (*M* = −0.356, *SE* = 0.407) did not show any significantly differences on the OxyHb contrast (*t* (1,6) = 0.315, *P* = 0.764, 95% CI [−1.53, 1.99], *g* = 0.193).

In addition, compared to the sham condition (*M* *= *3.814*, SE* = 1.212), females in the mPFC stimulation condition (*M* = −1.114, *SE* = 0.886) also had a significantly lower OxyHb contrast effect (*t* (1,9) = −2.797, *P* = 0.021, 95% CI [−8.91, −0.942], *g* = −1.602). In contrast, compared to the sham condition (*M* = −0.582, *SE* = 0.593), males in the mPFC stimulation condition (*M* = −1.154, *SE* = 0.950) did not show any significant differences on the OxyHb contrast (*t* (1,7) = 1.451, *P* = 0.190, 95% CI [−1.09, 4.57], *g* = 0.865).

Finally, no significant differences on Flanker OxyHb contrast were found between the dlPFC and mPFC conditions for either males (*t* (1,7) = −1.330, *P* = 0.225, 95% CI [−4.19, 1.17], *g* = −0.793) or females (*t* (1,10) = 0.324, *P* = 0.753, 95% CI [−1.90, 2.55], *g* = 0.183).

#### DD OxyHb contrast.

**Fig. 4.**
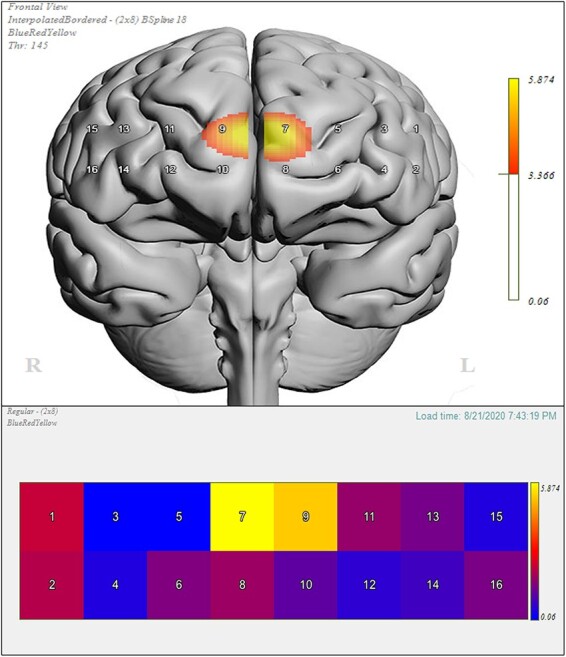
Left: Heat map of channels 1–16 illustrating the difference in OxyHb concentration between active iTBS (targeting left dlPFC and the bilateral dmPFC) and sham iTBS during the delay discounting task. Color coding was represented by the strength of the *F* and *P*-values; warmer colors represent stronger active and sham difference; darker shades of blue represent weak to no differences. Right: 3D anatomical overlay of the significant differences between active and sham. This figure was generated using fnirSoft (Version 4.11).

Following the calculation of the DD OxyHb effect (see Supplementary material), each channel was subject to a one-way ANOVA, to test for any hypothesized group differences (active iTBS and sham iTBS). Differences across channels are illustrated using a heat map and a 3D anatomical overlay (Figure 4), from corresponding *F*-values and *P-*values for each channel below.

**Fig. 5.**
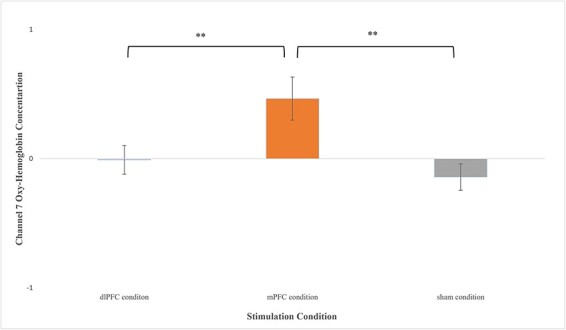
Means (±SE) for channel 7 OxyHb concentration for each treatment condition averaged across the three delay discounting blocks; (A) dlPFC condition (*M* = −0.00787, *SE* = 0.112 ), (B) mPFC condition (*M* = 0.466, *SE* = 0.167) and (C) sham condition (*M* = −0.142, *SE* = 0.103). **: *P* < 0.01.

**Fig. 6.**
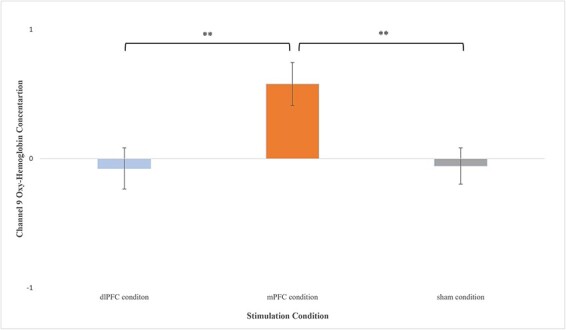
Means (±SE) for channel 9 OxyHb concentration for each treatment condition averaged across the three delay discounting blocks; (A) dlPFC condition (*M* = −0.0750, *SE* = 0.160), (B) mPFC condition (*M* = 0.579, *SE* = 0.167) and (C) sham condition (*M* = −0.0564, *SE* = 0.141). **: *P* < 0.01.

Analysis revealed significant task-related increases in OxyHb in channel 7 (*F*(2,34) = 5.874, *P* = 0.006, ${\eta _p}$^2^ = 0.257) and channel 9 (*F*(2,33) = 5.289, *P* = 0.010, ${\eta _p}$^2^ = 0.247) between active and sham iTBS. Variable means for both channels by stimulation condition are depicted in their respective figures below (Figures 5 and 6).

Planned comparisons for channel 7 indicated that compared to the sham condition, those in the mPFC stimulation condition had significantly higher task-related increases in OxyHb (*t* (1,21) = 3.154, *P* = 0.005, 95% CI [0.207, 1.01], *g* = 1.269) during the DD task (Figure 5). Similarly, compared to dlPFC condition, those in the mPFC condition had significantly higher task-related increases in OxyHb (*t* (1,23) = 2.437, *P* = 0.023, 95% CI [0.0717, 0.877], *g* = 0.950) during the DD task (Figure 5). On the other hand, compared to the sham condition, those in dlPFC condition had no significant differences in OxyHb concentrations (*t* (1,24) = 0.872, *P* = 0.392, 95% CI [−0.183, 0.453], *g* = 0.332) on the DD task.

A similar pattern was observed for channel 9; compared to the sham condition, those in the mPFC stimulation condition had significantly higher task-related increases in OxyHb (*t* (1,20) = 2.902, *P* = 0.009, 95% CI [0.179, 1.09], *g* = 1.190) during the DD task (Figure 6). Similarly, compared to the dlPFC stimulation condition, those in the mPFC stimulation condition had significantly higher task-related increases in OxyHb (*t* (1,23) = 2.796, *P* = 0.010, 95% CI [0.170, 1.14], *g* = 1.089) during the DD task (Figure 6). On the other hand, those in dlPFC stimulation condition did not show significant differences in task-related OxyHb compared to sham (*t* (1,23) = −0.085, *P* = 0.933, 95% CI [−0.474, 0.436], *g* = −0.033) during performance of the DD task.

In addition, two-way ANOVAs (stimulation × age category and stimulation × gender) were generated for both channels to determine if task-related OxyHb increases during the DD task were moderated by age category or gender. Results revealed no significant interaction effect between stimulation and age category or stimulation and gender for channels 7 and 9 (see Supplementary material for analysis).

Correction for false discovery rate (FDR; Benjamini & Hochberg, 1995) for the existing task-related OxyHb effects by channel revealed significant gender × stimulation effects for Channels 1 and 13 for the Flanker contrast, but the Channel 6 and 9 effects for the DD contrast did not survive FDR correction.

### Food consumption and task performance

#### Food consumption.

With respect to food consumption, the two-way (stimulation × age category) ANOVA revealed no significant main effect of stimulation (*F*(2,37) = 0.655, *P* = 0.526, ${\eta _p}$^2^= 0.034) or age category (*F*(1,37) = 3.068, *P* = 0.088, ${\eta _p}$^2^ = 0.077). The interaction between stimulation condition and age category was also not significant (*F*(2,37) = 1.231, *P* = 0.304, ${\eta _p}$^2^ = 0.062).

**Fig. 7.**
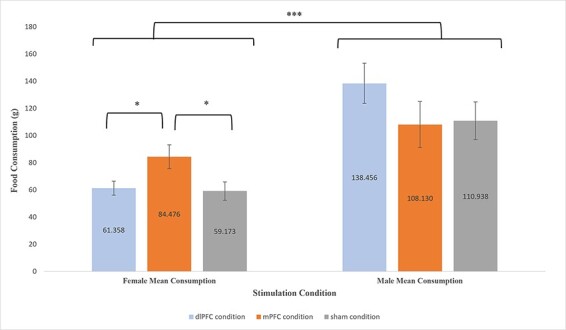
Mean (±*SE*) for food consumption (*g*) by gender for each treatment condition; (i) Females: (A) dlPFC condition (*M* = 61.358, *SE* = 5.143), (B) mPFC condition (*M* = 84.476, *SE* = 8.709) and (C) sham condition (*M* = 59.173, *SE *= 6.720); (ii) Males: (A) dlPFC condition (*M* = 138.456, *SE* = 14.766), (B) mPFC condition (*M* = 108.130, *SE* = 17.021) and (C) sham condition (*M* = 110.938, *SE* = 13.898). *: *P* < 0.05. ***: *P* < 0.001.

With respect food consumption, a two-way (stimulation × gender) ANOVA was conducted to examine the effect of treatment condition and gender on food consumption. The analysis revealed no significant main effect of stimulation (*F*(2,37) = 1.191, *P* = 0.315, ${\eta _p}$^2^ = 0.060), but a significant main effect of gender *F*(1,37) = 38.007, *P* < 0.001, $ {\eta _p}$^2^ = 0.507) on food consumption. The pattern of means suggests that across study conditions males (*M* = 119.174, *SE *= 8.951) consumed nearly twice as much food as females (*M* = 67.261, *SE* = 4.265). In addition, a statistically significant interaction was found between gender and stimulation group on food consumption (*F*(2,37) = 3.110, *P* = 0.040, ${\eta _p}$^2^ = 0.160), suggesting that the effect of stimulation on food consumption was significantly different for males and females. Variable means for all stimulation groups by gender are depicted in Figure 7.

Planned comparisons indicated that, compared to the sham condition (*M* *= *59.173*, SE* = 6.720), females in the mPFC condition (*M* = 84.476, *SE* = 8.709) consumed significantly more food (*t* (1,15) = 2.329, *P* = 0.034, 95% CI [2.14, 48.46], *g* = 1.074). In contrast, compared to the sham condition (*M* = 110.130, *SE* = 13.898), males in the mPFC condition (*M* = 108.130, *SE* = 13.898) did not consume significantly more food than females (*t* (1,8) = −0.128, *P* = 0.901, 95% CI [−53.48, 47.86], *g* = −0.073).

Compared to the females in the dlPFC condition (*M* *= *61.358*, SE* = 5.143), females in the mPFC condition (*M* = 84.476, *SE* = 8.709) consumed significantly more food (*t* (1,17) = −2.425, *P* = 0.027, 95% CI [−43.23, −3.00], *g* = −1.076). In contrast, males in the dlPFC condition did not significantly consume more food than males in the mPFC condition (*t* (1,8) = 1.346, *P* = 0.215, 95% CI [−21.64, 82.29], *g* = 0.768). Overall, no significant differences in food consumption were found between those in dlPFC condition and sham condition for both males (*t* (1,8) = 1.357, *P* = 0.212, 95% CI [−19.24, 74.27], *g* = 0.775) and females (*t* (1,18) = 0.263, *P* = 0.796, 95% CI [−15.28, 19.66], *g* = 0.113).

Additional analyses were conducted for each food type separately: potato chips (salty) and chocolate (sweet). Two-way ANOVA was performed to determine if there were any main effects or interactions between our categorical variables of interest: stimulation, age category and gender (see Supplementary material). Analysis revealed that chocolate consumption appear to be driving the stimulation-induced gender effect. (see Supplementary material).

#### Cognitive task performance.

Two-way ANOVA (stimulation × age category and stimulation × gender) was generated to examine the main effects of stimulation group (active dlPFC and active mPFC and sham stimulation) on Flanker inference score and log transformed DD (*k* values), as well as the interaction effects on each of variable. Results revealed neither significant main stimulation effect nor interaction effect between stimulation × age category or stimulation × gender on both cognitive tasks (see Supplementary material).

## Discussion

The purpose of the current study was to examine the effects of excitatory brain stimulation (iTBS) on calorie-dense food consumption and to examine potential demographic moderators of any such effects. Two prefrontal stimulation targets—the dlPFC and the dmPFC—were of *a-priori* interest because of their differential roles in behavioral inhibition and evaluative processing, respectively. Findings revealed reliable effects of iTBS on region-specific, task-related changes in neural activity: excitatory stimulation targeting the left dlPFC resulted in a neural efficiency effect in the lateral PFC in response to an interference task, whereas excitatory stimulation targeting the medial PFC resulted in greater neuronal recruitment in response to an evaluative processing task. These effects were theoretically meaningful and confirm the presence of a stimulation effect on the underlying neural substrates being targeted; the latter is an important finding, as relatively little validation of iTBS effects on the cortex have been undertaken in single stimulation-session format, and no prior studies comparing lateral and medial PFC stimulation using iTBS within the same study exist to our knowledge.

With respect to eating outcomes, findings were mixed. A significant effect of iTBS on eating was evident for females in the dmPFC stimulation condition such that female participants consumed significantly more calorie dense snack foods following active iTBS than following sham stimulation. The observed iTBS-induced increase in consumption suggested that enhancement of evaluative processing may have stimulated appetite; such a finding could reflect an amplification of context-induced bias of evaluative processing in the direction of indulgent eating because the presence of generally appetitive foods and a permissive eating environment (e.g. encouragement ‘to eat as much as you like’ to make ratings). An alternative explanation might be that excitatory stimulation of the dmPFC could have generated more spontaneous mind wandering about appetitive dimensions of the food via activation of the default mode network (Horn *et al.*, 2014), which would be impelling of consumption particularly under conditions of fasting (as was the case for our participants). Granular analysis by food sub-type revealed that these eating effects were mostly driven by the consumption of sweet snack foods rather than salty snack foods, which may be consistent with this hypothesis.

Gender differences may have emerged due to differences in social cognition and cue sensitivity between males and females. It is well documented that females are prone to higher levels of everyday dietary restraint than men (Rolls *et al.*, 1991; Wardle *et al.*, 2004; Darcy *et al.*, 2013). For this reason, females may have experienced relatively more potentiated evaluative processing in relation to tempting food cues in the eating situation presented. Additionally, fMRI studies have demonstrated higher reactivity in craving and taste-related brain regions to appetitive food cues in women compared to men (Uher *et al.*, 2006; Frank *et al.*, 2010; Atalayer *et al.*, 2014). Lastly, the eating effect driven by chocolate consumption in females can be explained by previous literature that has identified woman reporting stronger cravings for cues to sweet foods than savory foods, when compared to men (Zellner *et al.*, 1999; Hallam *et al.*, 2016).

In terms of contribution to the existing research literature, this is the only experimental study to our knowledge that has employed iTBS targeting the dmPFC to assess impact on eating behavior in the laboratory setting. One prior case study (Downar *et al.*, 2012) reported that high frequency excitatory rTMS targeting the dmPFC increased the ability to control bingeing in bulimia. This perhaps suggests differences in excitatory stimulation effects on the dmPFC between healthy and vulnerable individuals could be due to differences in frontal-striatal activity (Downar *et al.*, 2012). It should be noted also that the study in question employed a multi-session stimulation and did not specifically use iTBS. As such, there is ample justification for future research to further explore the effects of neuromodulation of the mPFC and its impact on eating.

The fNIRS data presented in this study highlight the value added by combining neuroimaging techniques with neuromodulation when examining PFC function in eating research. The findings presented are in agreement with previous studies that have shown neural recruitment of the lateral areas of the PFC to be important for inhibitory/anticipatory mentally effortful tasks (Vassena *et al.*, 2019; Curtin *et al.*, 2019a), whereas activation of medial PFC has been observed during tasks involving relative weighing of values attached to temporally proximal and distal outcomes (Marco-Pallarés *et al.*, 2010; Mitchell *et al.*, 2011; Wang *et al.*, 2014).

The lack of significant behavioral effects on both cognitive tasks can be partially explained in part due to the reliability of behavioral iTBS effects across neuromodulation studies. For example, studies examining the effects of iTBS targeting the dlPFC on executive functioning tasks have been mixed and more variable across specific tasks than cTBS (Lowe *et al.*, 2018a). It could be the case that iTBS only achieves consistent excitatory effects at the behavioral (i.e. task performance) level, when stimulation effects on neuronal populations are aggregated across several stimulation sessions over extended periods of time. For example, there is evidence from previous studies that multi-session excitatory rTMS can improve executive function and obesity (Kim *et al.*, 2012; Gaudeau-Bosma *et al.*, 2013; Li *et al.*, 2017). Likewise, a previous study administering multi-session iTBS to the left dlPFC among patients with medication-resistant depression revealed a significant improvement on executive task performance compared to sham (Cheng *et al.*, 2016). In relation to intertemporal choice, the current study contributes to a growing evidence (Cho *et al.*, 2010; Yang *et al.*, 2018) of the possibility that iTBS targeting the PFC may not be able to alter cognitive impulsivity, at the behavioral level, within a single session. Overall, due to the small number of studies utilizing iTBS protocols (Lowe *et al.*, 2018a), it would be of further interest to conduct additional research in validating the efficacy of iTBS on the improvement of executive functioning and its impact on cognitive impulsivity.

Key strengths of this study include the integration of neuroimaging methods with neuromodulation, the use of blinding combined with sham coil for the sham condition and the targeting of multiple PFC subregions within the same study. Likewise, the use of a behavioral test of actual food consumption is an important improvement over studies that employ only self-reported measurement of food cravings or abstract choice paradigms that involve no actual food consumption. Given the role of cortical nodes in choice implementation—beyond simply choice making—the latter is an important consideration in social neuroscience research involving eating. Focusing only on decision-making paradigms may lead to a less than complete picture of how and when particular PFC subregions participate in eating behavior in the real world. Finally, the mPFC is rarely explored as a stimulation target in eating behavior research, and the current study helps to add to this new area of inquiry. Limitations of this study include the relatively modest sample size (though typical of neuromodulation studies), which may have decreased the statistical power for the behavioral measurements. Other limitations of this study include the lack of double blinding and uncertain generalizability; however, our sample did employ a considerably larger age range than most eating behavior studies involving the use of rTMS and/or fNIRS.

Finally, the findings presented in relation to the effects of iTBS on eating are novel and should be verified in a larger and more diverse sample and with other imaging modalities (e.g. EEG and fMRI). The findings with respect to the neural effects—quantified by fNIRS—should similarly be interpreted with caution and treated primarily as a descriptive validation of the stimulation effects (i.e. a manipulation check) given the non-correction for multiple comparisons. The relatively modest sample size may make spurious findings more likely under such conditions. More highly powered studies may provide more confidence in the reliability of TBS effects on the specific prefrontal subregions examined in the context of the current study.

## Conclusions

The findings from this study suggest that there is a considerable amount of complexity in iTBS effects on eating. Specifically, stimulation effects might be modified by PFC subregion and participant gender, as well as the relative valence of contextual cues. Findings confirmed expected effects of medial and lateral PFC stimulation on neuronal substrates but identified a pattern of amplified calorie-dense food consumption among those in the mPFC condition compared to sham. This effect was moderated by participant gender but was relatively invariant across the age span. The nature of the observed findings may reflect the generally permissive eating environment, which encouraged indulgence. Future studies would benefit from exploring the range of mPFC stimulation effects that could be produced in inhibitory or facilitating eating environments and using different variants of TBS protocols (e.g. cTBS). Such studies focusing on the dlPFC have produced interesting findings (Safati and Hall, 2019) and could provide a template for further research involving the mPFC.

## Supplementary Material

nsab023_Supp
