# Erratum to: Effects of prefrontal theta burst stimulation on neuronal activity and subsequent eating behavior: an interleaved rTMS and fNIRS study

**DOI:** 10.1093/scan/nsab040

**Published:** 2021-04-08

**Authors:** Idris Fatakdawala, Hasan Ayaz, Adrian B Safati, Mohammad N Sakib, Peter A Hall

**Affiliations:** School of Public Health and Health Systems, University of Waterloo, Waterloo, ON, Canada; School of Biomedical Engineering, Science and Health, Drexel University, Philadelphia, PA, USA; Department of Psychology, College of Arts and Sciences, Drexel University, Philadelphia, PA, USA; Drexel Solutions Institute, Drexel University, Philadelphia, PA, USA; Department of Family and Community Health, University of Pennsylvania, Philadelphia, PA, USA; Center for Injury Research and Prevention, Children’s Hospital of Philadelphia, Philadelphia, PA, USA; School of Public Health and Health Systems, University of Waterloo, Waterloo, ON, Canada; School of Public Health and Health Systems, University of Waterloo, Waterloo, ON, Canada; School of Public Health and Health Systems, University of Waterloo, Waterloo, ON, Canada; Centre for Bioengineering and Biotechnology, University of Waterloo, Waterloo, Canada; Department of Psychology, University of Waterloo, Waterloo, ON, Canada

The figures in this paper erroneously did not include the bars indicating significant differences between groups. The affected figures have been corrected online.

