# Supplementary material for: Effects of prefrontal theta burst stimulation on neuronal activity and subsequent eating behavior: an interleaved rTMS and fNIRS study
Source: Soc Cogn Affect Neurosci. 2021 Feb 22;18(1):nsab023. doi: 10.1093/scan/nsab023 (PMC10074772; doi:10.1093/scan/nsab023)
Supplement: nsab023_Supp [file nsab023_supp.zip › Supplementary Material.docx]

**Supplementary Material**

Effects of prefrontal theta burst stimulation on neuronal activity and subsequent eating behavior: an interleaved rTMS and fNIRS study

Idris Fatakdawala^a^, Hasan Ayaz^b,c,d,e,f^, Adrian Safati^a^, Mohammad Nazmus Sakib^a^ & Peter A. Hall^a,g*^

^a^ School of Public Health & Health Systems, University of Waterloo, Ontario, Canada

^b^ School of Biomedical Engineering, Science and Health, Drexel University, Philadelphia PA, USA

^c^ Department of Psychology, College of Arts and Sciences, Drexel University, Philadelphia, PA, USA

^d^ Drexel Solutions Institute, Drexel University, Philadelphia, PA, USA

^e^ Department of Family and Community Health, University of Pennsylvania, Philadelphia, PA, USA

^f^ Center for Injury Research and Prevention, Children’s Hospital of Philadelphia, Philadelphia, PA, USA

^g^ Centre for Bioengineering and Biotechnology, University of Waterloo, Waterloo, Ontario, Canada.

**Flanker Oxy-Hemoglobin Contrast Effect Calculation**

The Flanker oxy-hemoglobin contrast effect for each fNIRS channel was calculated by subtracting the mean hemodynamic response in the congruent condition (obtained by collapsing individual mean hemodynamic responses from the two congruent blocks) from the mean hemodynamic response in incongruent condition (obtained by collapsing individual mean hemodynamic responses from the two incongruent blocks):

*Incongruent/Congruent Contraste Effect =*

*(CHn_INC1 + CHn_INC2) – (CHn_CON1 + CHn_CON2)*

where,

*CHn_INC1* refers to the mean hemodynamic response for incongruent task #1 for channel *n*

*CHn_INC2* refers to the mean hemodynamic response for incongruent task #2 for channel *n*

*CHn_CON1* refers to the mean hemodynamic response for congruent task #1 for channel *n*

*CHn_CON2* refers to the mean hemodynamic response for congruent task #2 for channel *n*

**Delay Discounting Oxy-Hemoglobin Calculation**

The delay discounting oxy-hemoglobin concentration for each fNIRS channel was calculated by taking the average of mean hemodynamic responses across the three delay discounting tasks for each channel:

*Delay Discounting Oxy-Hemoglobin:*

*(CHn_DD1 + CHn_DD2 + + CHn_DD3)/3*

where,

*CHn_DD1* refers to the mean hemodynamic response for the delay discounting task #1 for channel *n*

*CHn_DD2* refers to the mean hemodynamic response for the delay discounting task #2 for channel *n*

*CHn_DD3* refers to the mean hemodynamic response for the delay discounting task #3 for channel *n*

**Delay Discounting Oxy Hemoglobin Concentration (Interaction Analysis)**

*Channel 7*

With respect to delay discounting oxy-hemoglobin concentrations, the two-way (stimulation x age category) ANOVA revealed a significant main effect of stimulation (*F*(2,31) = 5.370, *p* = .010, $\eta_{p}$^2^= 0.257), but no significant main effect of age category (*F*(1,31) = 1.032, *p* = .318, $\eta_{p}$^2^= 0.032). The interaction between stimulation condition and age category was not significant (*F*(2,31) = .518, *p* = .601, $\eta_{p}$^2^= 0.032).

With respect to delay discounting oxy-hemoglobin concentrations, the two-way (stimulation x gender) ANOVA revealed a significant main effect of stimulation (*F*(2,31) = 5.159, *p* = .012, $\eta_{p}$^2^ = .250), but no significant main effect of gender (*F*(1,31) = .260, *p* = .614, $\eta_{p}$^2^= 0.008). The interaction between stimulation condition and gender was not significant (*F*(2,31) = .700, *p* = .504, $\eta_{p}$^2^ = 0.043).

*Channel 9*

With respect to delay discounting oxy-hemoglobin concentrations, the two-way (stimulation x age category) ANOVA revealed a significant main effect of stimulation (*F*(2,30) = 4.573, *p* = .016, $\eta_{p}$^2^ = 0.241), but no significant main effect of age category (*F*(1,30) = .341, *p* = .564, $\eta_{p}$^2^ = 0.011). The interaction between stimulation condition and age category was found not to be significant (*F*(2,30) = 1.424, *p* = .257, $\eta_{p}$^2^ = 0.087).

With respect to delay discounting oxy-hemoglobin concentrations, the two-way (stimulation x gender) ANOVA revealed a significant main effect of stimulation (*F*(2,30) = 6.587, *p* = .004, $\eta_{p}$^2^ = 0.305), but no significant main effect of gender (*F*(1,30) = 1.323, *p* = .259, $\eta_{p}$^2^ = 0.042). The interaction between stimulation condition and gender was not significant (*F*(2,30) = 2.219, *p* = .126, $\eta_{p}$^2^ = 0.129).

**Granular Food Choice Analyses**

*Total Potato Chips Consumption*

With respect to potato chips consumption, a two-way (stimulation x age category) ANOVA revealed no significant main effect of stimulation (*F*(2,37) = 1.850, *p* = 0.171, $\eta_{p}$^2^ = 0.091) and age category (*F*(1,37) = 3.733, *p* = 0.061, $\eta_{p}$^2^ =0.092). The interaction between stimulation condition and age category was also not significant (*F*(2,37) = 1.041, *p* = 0.363, $\eta_{p}$^2^ = 0.053).

A two-way (stimulation x gender) ANOVA revealed no significant main effect of stimulation (*F*(2,37) = 1.265, *p* = 0.294, $\eta_{p}$^2^= 0.064), but a significant main effect for gender (*F*(1,37) = 8.158, *p* = 0.007, $\eta_{p}$^2^= 0.181). Males (*M* = 49.023, *SE* = 5.368) consumed significantly more salty foods than females (*M* = 31.615, *SE* = 3.104) across stimulation conditions. The interaction between stimulation condition and gender was not significant (*F*(2,37) = .091, *p* = 0.913, $\eta_{p}$^2^= 0.005). Variable means for all study conditions by gender are depicted in Figure S1.

**Figure S1**. Mean (+/-SE) for total potato chips consumption (g) by gender for each treatment condition; i) Females: a) dlPFC condition (M = 25.458, SE = 4.649), b) mPFC condition (M = 39.811, SE = 5.228) and c) sham condition (M = 31.854, SE = 5.797); ii) Males: a) dlPFC condition (M = 44.872, SE = 5.741), b) mPFC condition (M = 53.190, SE = 11.608) and c) sham condition (M = 49.008, SE = 11.232). ** p < .01.

*Total Chocolate Consumption*

With respect to chocolate consumption, a two-way (stimulation x age category) ANOVA revealed no significant main effects of stimulation (*F*(2,37) = 1.171, *p* = 0.321, $\eta_{p}$^2^=0.060) or age category (*F*(2,37) = 0.841, *p* = 0.365, $\eta_{p}$^2^= 0.022). The interaction between stimulation condition and age category was also not significant (*F*(2,37) = 2.544, *p* = 0.092, $\eta_{p}$^2^= 0.121).

A two-way (stimulation x gender) ANOVA revealed a significant main effect of stimulation (*F*(2,37) = 4.574, *p* = 0.017, $\eta_{p}$^2^= 0.198) and gender (*F*(1,37) = 33.136, *p* = < 0.001, $\eta_{p}$^2^= 0.472), such that those in the active stimulation conditions (dlPFC : *M* = 53.293, *SE* = 7.976, mPFC: *M* = 47.296, SE = 5.169) consumed more than those in the sham stimulation condition (*M* = 39.602, *SE* = 5.952). The results also suggest that males (*M* = 68.249, *SE* = 7.342) overall consumed more sweet foods than females (*M* = 35.651, *SE* = 2.717) regardless of treatment condition.

The interaction between stimulation condition and gender was also significant (*F*(2,37) = 6.547, *p* = .004. $\eta_{p}$^2^= 0.255)**,** suggesting that the effect of stimulation was significantly different for males and females**.** Variable means for all study conditions by gender are depicted in Figure S2.

***

*

**Figure S2**. Mean (+/-SE) for total chocolate consumption (g) by gender for each treatment condition; i) Females: a) dlPFC condition (M = 35.900, SE = 3.515), b) mPFC condition (M = 44.665, SE = 6.962) and c) sham condition (M = 27.332, SE = 1.815); ii) Males: a) dlPFC condition (M = 91.554, SE = 12.768), b) mPFC condition (M = 51.506, SE = 8.093) and c) sham condition (M = 61.688, SE = 10.995). *: p < .05. ***: p < .001.

Planned comparisons indicated that compared to the sham condition (*M* = 27.332, *SE* = 1.815), those females in the mPFC condition (*M* = 44.665, *SE* = 6.962) consumed significantly more sweet foods (*t* (1,8) = 2.409, *p* = 0.043, 95% CI [0.725, 33.94], *g* = 1.173). Levene’s test indicated unequal variances (*F* = 10.696, *p* = 0.005), so the degrees of freedom were adjusted from 15 to 8.

There was also a marginally significant difference in the consumption of sweet foods between females in the dlPFC condition (M = 35.900, SE = 3.515) and sham condition (*t* (1,15) = 2.166, *p* = 0.058, 95% CI [0.124, 17.01], *g* = .871). Levene’s test indicated unequal variances (*F* = 11.481, *p* = 0.003), so the degrees of freedom were adjusted from 18 to 15.

Among males, it was found that those in the mPFC condition (*M* = 51.506*, SE* = 8.093) did not consume significantly more (*t* (1,8) = -.746, *p* = 0.477, 95% CI [-41.66, 21.30], *g* = -.426), food than those in the sham condition (*M* = 61.688, *SE* = 10.995). In addition, there were no significant differences in the consumption of sweet foods between those in the dlPFC and sham condition for males (*t* (1,8) = 1.773, *p* = 0.114, 95% CI [-8.99, 68.72], *g* = 1.012).

Lastly, among males it was found that those in dlPFC condition (M = 91.554, SE = 12.768) consumed more chocolate than those in the mPFC condition (*t* (1,8) = 2.649, *p* = 0.029, 95% CI [5.19, 74.91], *g* = 1.512). In contrast, no significant differences in chocolate consumption were found between the dlPFC and mPFC for females (*t* (1,17) = -1.249, *p* = 0.240, 95% CI [-23.94, 6.41], *g* = -.541).

**Cognitive Tasks**

*Flanker Interference Scores*

With respect to flanker interference scores, the two-way (stimulation x age category) ANOVA revealed no main effect of stimulation (*F*(2,36) = 0.706, *p* = 0.500, $\eta_{p}$^2^= 0.038) and no significant main effect of age category (*F*(1,36) = 0.278, *p* = 0.601, $\eta_{p}$^2^= 0.008 ). The interaction between stimulation condition and age category was also not significant (*F*(2,36) = 1.263, *p* = 0.295, $\eta_{p}$^2^= 0.066).

With respect Flanker interference scores, a two-way (stimulation x gender) ANOVA was conducted to examine the effect of treatment condition and gender on Flanker interference scores. The analysis revealed no significant main effect of stimulation (*F*(2,36) = 0.706, *p* = 0.500, $\eta_{p}$^2^= 0.038) or gender (*F*(1,36) = 2.197 , *p* = 0.147, $\eta_{p}$^2^= 0.058) on Flanker performance. The interaction between stimulation and gender was also not significant (*F*(2,36) = 0.119, *p* = 0.888, $\eta_{p}$^2^= 0.007). Variable means for all study conditions by gender are depicted in Figure S3.

**Figure S3**. Mean (+/-SE) for Flanker interference score (ms) by gender for each treatment condition; i) Females: a) dlPFC condition (M = 50.972, SE = 9.168), b) mPFC condition (M = 62.669, SE = 16.865) and c) sham condition (M = 54.258, SE = 10.849); ii) Males: a) dlPFC condition (M = 60.489, SE = 9.828), b) mPFC condition (M = 82.124, SE = 20.126) and c) sham condition (M = 76.576, SE = 17.199).

*Log Transformed Average Delay Discounting (k values)*

With respect to log transformed delay discounting *k* values, the two-way (stimulation x age category) ANOVA revealed no main effect of stimulation (*F*(2,37) = 0.043, *p* = 0.958, $\eta_{p}$^2^= 0.002 ) and no significant main effect of age category (*F*(1,37) = 2.684, *p* = 0.110, $\eta_{p}$^2^= 0.068). The interaction between stimulation condition and age category was also not significant (*F*(2,37) = 0.192, *p* = 0.826, $\eta_{p}$^2^= 0.010).

With respect to log transformed delay discounting *k* values, a two-way (stimulation x gender) ANOVA was conducted to examine the effect of treatment condition and gender on log transformed delay discounting *k* values. The analysis revealed no significant main effect of stimulation (*F*(2,37) = 0.083, *p* = 0.921, $\eta_{p}$^2^= 0.004), and gender *F*(1,37) = 0.90 , *p* = 0.766, $\eta_{p}$^2^= 0.002) on log transformed delay discounting *k* values. The interaction term between stimulation condition and gender was not significant (*F*(2,37) = 0.181, *p* = 0.835, $\eta_{p}$^2^= 0.010). Variable means for all study conditions by gender have been graphed by taking the absolute value of the log transformed *k* values in Figure S4.

.

**Figure S4**. Mean (+/-SE) for Log10 transformed averaged delay discounting k values by gender for each treatment condition; i) Females: a) dlPFC condition (M = 2.253, SE = 0.246), b) mPFC condition (M = 2.168, SE = 0.210) and c) sham condition (M = -2.281, SE = 0.243); ii) Males: a) dlPFC condition (M = 2.013, SE = 0.306), b) mPFC condition (M = 2.275, SE = 0.357) and c) sham condition (M = -2.203, SE = 0.339).
